# Comparative effectiveness of N95 respirators and surgical/face masks in preventing airborne infections in the era of SARS-CoV2 pandemic: A meta-analysis of randomized trials

**DOI:** 10.1371/journal.pone.0242901

**Published:** 2020-12-15

**Authors:** Katarzyna Barycka, Lukasz Szarpak, Krzysztof Jerzy Filipiak, Milosz Jaguszewski, Jacek Smereka, Jerzy Robert Ladny, Oguz Turan

**Affiliations:** 1 Polish Society of Disaster Medicine, Warsaw, Poland; 2 Maria Sklodowska-Curie Medical Academy in Warsaw, Warsaw, Poland; 3 First Chair and Department of Cardiology, Medical University of Warsaw, Warsaw, Poland; 4 First Department of Cardiology, Medical University of Gdansk, Gdansk, Poland; 5 Department of Emergency Medical Service, Wroclaw Medical University, Wroclaw, Poland; 6 Clinic of Emergency Medicine, Medical University of Bialystok, Bialystok, Poland; 7 Department of Outcomes Research, Anesthesiology Institute, Cleveland Clinic, Cleveland, Ohio, United States of America; South African Medical Research Council, SOUTH AFRICA

## Abstract

**Background:**

Recently, several randomized controlled trials (RCTs) have evaluated the effect of N95 respirators compared with medical masks to protect against acute respiratory infections. However, these studies are limited by modest sample sizes and inconclusive results. Therefore, the goal of the present study was to review the relevant and available published RCTs with the aid of the increased power of meta-analytic methods in order to assess the effectiveness of medical masks and N95 respirators in reducing the risk of respiratory infections.

**Methods:**

This meta-analysis follows the recommendations of the Preferred Reporting Items for Systematic Reviews and Meta-Analyses (PRISMA) statement for conducting and reporting results. We searched PubMed, Web of Science, Embase, and Cochrane databases from inception through April 1, 2020 to identify potentially relevant studies. Two authors (LS and JS) independently searched the titles and abstracts of the potentially eligible articles. They independently retrieved required data from the eligible trials; the data were initially tabulated for statistical analysis. Two authors (JRL and LS) independently assessed the methodological quality of the included RCTs using the Cochrane Collaboration’s tool for assessing risk of bias.

**Results:**

Six articles met the inclusion criteria. The pooled analysis showed that N95 respirators did not reduce the risk of infection with respiratory viruses compared with medical/surgical masks (5.7% vs. 7.9%; RR = 1.12; 95% CI: 0.88–1.41; p = 0.36); however, there was no statistically significant difference in laboratory-confirmed influenza between N95 and medical masks (RR = 0.91; 95% CI: 0.77–1.07; p = 0.26). Medical masks provided similar protection against other viruses, including coronavirus (RR = 0.74; 95% CI: 0.32–1.73; p = 0.49). Respiratory illness, as well as influenza-like illness were less frequently observed with N95 respirators.

**Conclusions:**

Our meta-analysis suggests that there are insufficient data to definitively determine whether N95 respirators are superior to medical masks in protection against transmissible acute respiratory infections. Further randomized trials are necessary to compare the above methods of respiratory protection in the context of COVID-19 incidence.

## Background

The COVID-19 pandemic has now affected most countries in the world. The SARS-CoV-2 infection is droplet-transmitted and poses a serious threat to public health globally because of the high number of infections, including a high incidence of severe respiratory failure. In many regions, acute respiratory distress syndrome is leading to depletion of resources and capacities of the healthcare systems [1]. Clinically, the COVID-19 pandemic presents with coughing, dyspnea, fever, headache, sore throat, and loss of smell. However, serious complications of COVID-19 can occur after disease progression, the most common major complication is Acute Respiratory Distress Syndrome or also known as ARDS. This clinical feature is one of the reasons the COVID-19 pandemic became infamous for its requirement of mechanical ventilation. However, further systemic complications such as cardiac arrhythmia as well as other immunologic and thromboembolic events. It is thus essential to use appropriate protection, including personal protective equipment (PPE), of which a suitable respirator, such as those rated N95, is a crucial component [2]. No specific treatment is yet available for COVID-19, although work is underway on the use of previously known drugs such as chloroquine and its derivatives or antivirals. Currently many centers are working on the development of a vaccine but at the moment, prevention and isolation are of primary importance.

Recently, several randomized controlled trials (RCTs) have evaluated the effect of N95 respirators compared with medical masks to protect against acute respiratory infections. An N95 respirator or N95 mask is a mechanical filter respirator that is designed to meet the NIOSH N95 classification of air filtration, filtering at least 95% of airborne particles with a penetrating aerosol size of 0.3 μm. They have to adhere strictly to 42 CFR Part 84 regulations in order to be able to be considered an official N95. In contrast, medical masks also known as surgical masks are designed to be more fluid resistance and to protect the wearer against fluid penetration. However, surgical masks are not designed to be and are prohibited from being labelled as antimicrobial or antiviral protection or particulate filtration. Surgical masks instead have to adhere to a different standard, ASTM F1862, which regulates the fluid impact amount, velocity, and viscosity. During the COVID-19 pandemic, the demand for both of these kinds of masks increased exponentially, resulting in shortages across all hospital systems. The global spread of the epidemic combined with the massive scale served to deplete existing stockpiles as well as greatly hinder the production of new respirators. During the epidemic, drastic steps were taken to order new respirators as well as even attempt to sterilize previously used respirators in order to increase the effective size of the existing stockpiles.

However, these studies are limited by modest sample sizes and inconclusive results [3–8]. Therefore, the goal of the present study is to review the relevant and available published RCTs with the aid of the increased power of meta-analytic methods in order to test the hypothesis that, compared with medical masks N95 respirators would reduce the risk of respiratory infections.

## Methods

This meta-analysis follows the recommendations of the Preferred Reporting Items for Systematic Reviews and Meta-Analyses (PRISMA) statement for conducting and reporting results [9]. The protocol of this meta-analysis has not been registered.

### Eligibility criteria

The meta-analysis included published RCTs comparing medical/surgical masks (MSMs) with N95 respirators to protect against acute respiratory infections. Case-control studies, non-randomized studies, trials conducted on simulated models, editorials, reviews, guidelines, and theoretical models were excluded from the review.

### Information sources and search strategy

We searched PubMed, Web of Science, Embase, and Cochrane databases from inception through to April 1, 2020 in order to identify potentially relevant studies. The search terms included: “influenza” OR “coronavirus” OR “virus” OR “COVID-19” OR “SARS-CoV-2” OR “SARS-CoV” OR “SARS” OR “MERS” OR “acute respiratory tract infection” OR “acute respiratory infection” AND “masks” OR “respiratory protective device” OR “personal protect” OR “personal protective equipment” OR “medical mask” OR “surgical mask” OR “facemask” OR “N95” OR “respirators”. The search was performed with no language restriction. Reference lists of relative articles were also reviewed.

### Study selection

Two authors (LS and JS) independently searched the titles and abstracts of the potentially eligible articles. Furthermore, full texts of the possible articles were retrieved and assessed for eligibility. Any disputes between the two authors were solved by discussion and consultation with a third author (JRL).

### Data collection process

Two authors (LS and JS) independently retrieved the required data from the eligible trials; the data was initially tabulated in a Microsoft Excel^TM^ (Microsoft Corp., Redmond, WA, USA) data set. Another author (KJF) cross-checked the data before analysis.

### Data items

The following data was retrieved from the full texts of all studies: first author, year of publication, sample size, characteristics of participants, type of mask, laboratory-confirmed infection with any respiratory virus, laboratory-confirmed bacterial colonization, laboratory-confirmed influenza (including influenza A and B) as well as other respiratory viruses, respiratory illness, influenza-like illness, and work absence.

### Risk of bias in the included studies

Two authors (JRL and LS) independently assessed the methodological quality of the included RCTs using the Cochrane Collaboration’s tool for assessing risk of bias [10]. The following domains were assessed: random sequence generation, allocation concealment, blinding of participants and personnel, blinding of outcome measurement, incomplete outcome data, and selective reporting. For each domain of bias, the trials were classified as representing low, unclear, or high risk of bias.

### Statistical analysis

Statistical heterogeneity was assessed with the I^2^ methodology. Values of I^2^ > 50% and > 75% were considered to indicate moderate and significant heterogeneity among studies, respectively [11]. Because of possible clinical heterogeneity due to study design and participant population, we used a fixed effect model for all pooled analyses. The pooled effect estimates for binary variables were expressed as relative risk (RR). All statistical variables were calculated with 95% confidence interval (CI). All p-values were two-tailed and considered statistically significant if p < 0.05. Publication bias was evaluated by visually inspecting the funnel plots. All statistical analyses were performed with the Review Manager software, version 5.3 (RevMan, Cochrane Collaboration, Oxford, UK).

## Results

### Trial identification and characteristics

**Fig 1:**
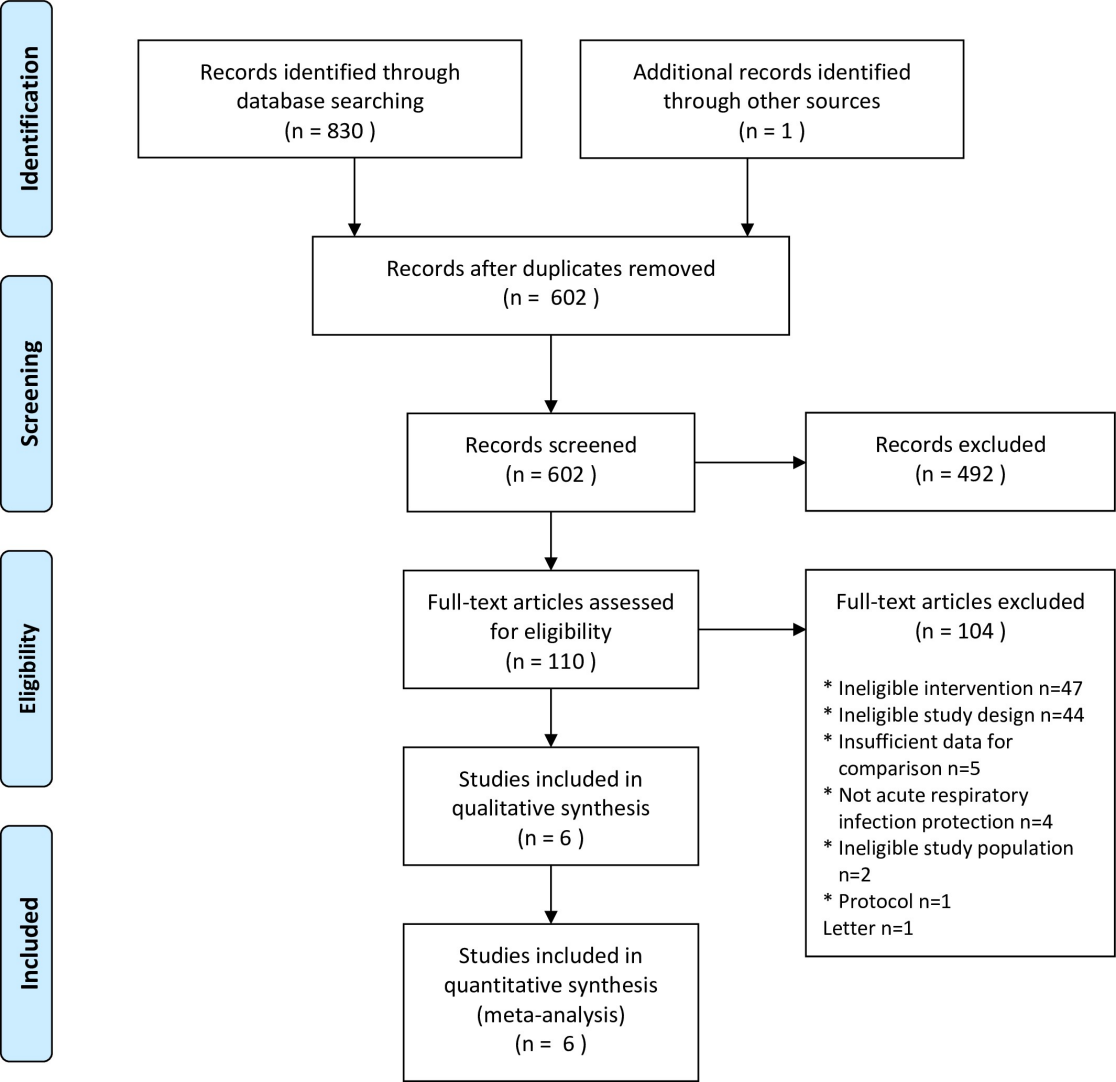
Flow diagram showing stages of database searching and study selection as per PRISMA guidelines.

The literature search yielded 831 records, and six multi-center RCTs fulfilling inclusion criteria were eligible for final analysis [3–8]. The overview of the study selection process is presented in Fig 1. The Cochrane risk of bias score varied across the trials (S1 and S2 Figs). The main characteristics of the included studies are shown in Table 1. Three of them were conducted in China [5–7], one in Canada [3], one in Australia [4], and one in the USA [8].

**Table 1:** Characteristics of included studies.

Study / Year	Country	Study design	Setting	Type of subject	Disease caused by virus	No. of participants	Interventions	Outcome(s)
Loeb et al. /2004	Canada	Cohort study	2 hospitals: coronary care units and ICUs with SARS patients	Nurses	SARS	43	Intervention: N95 respirator	Laboratory-confirmed respiratory infection
							Control: surgical mask	
Loeb et al. /2009	Canada	RCT–individual-level randomization	8 hospitals: ED, acute medical units and pediatric units	Nurses	Influenza A and B, respiratory syncytial virus metapneumovirus, parainfluenza virus, rhinovirus—enterovirus, coronavirus, adenovirus	446	Intervention: targeted fit-tested N95 respirator	Laboratory-confirmed infection, influenza-like illness, workplace absenteeism; 5wk follow-up
							Control: surgical mask	
MacIntyre et al. /2009	Australia	RCT—Cluster randomization by hospital	145 households	Households	Influenza A and B, respiratory syncytial virus metapneumovirus, parainfluenza virus, rhinovirus—enterovirus, coronavirus, adenovirus	186	Intervention 1: continual medical mask	Laboratory-confirmed infection, influenza-like illness; 2wk follow-up
							Intervention 2: continual nonfit-tested N95 respirator	
							Control: lifestyle measures	
MacIntyre et al. /2011/2014	China	RCT—Cluster randomization by hospital	15 hospitals: ED and respiratory wards	Healthcare workers	Influenza A and B, respiratory syncytial virus metapneumovirus, parainfluenza virus, rhinovirus—enterovirus, coronavirus, adenovirus	1441 nurses	Intervention 1: continual fit-tested N95 respirator	Laboratory-confirmed infection, influenza-like illness; 5wk follow-up
							Intervention 2: continual nonfit-tested N95 respirator u	
							Control: continual surgical mask	
MacIntyre et al. 2013	China	RCT—Cluster randomization by ward	19 hospitals: ED and respiratory wards	Healthcare workers	Influenza A and B, respiratory syncytial virus metapneumovirus, parainfluenza virus, rhinovirus 0 enterovirus, coronavirus, adenovirus	1669	Intervention 1: continual fit-tested N95 respirator use	Laboratory-confirmed infection, influenza-like illness; 5wk follow-up
							Intervention 2: targeted fit-tested N95 respirator use	
							Control: continual surgical mask use	
Radonovich et al. /2019	USA	RCT—Cluster randomization by outpatient clinic or outpatient setting	7 hospitals: primary care facilities, adult and pediatric clinics, dialysis units, urgent care facilities and ED, and emergency transport services	Healthcare workers	Coxsackie/echoviruses; coronaviruses: HKU1, NL63, OC43, 229R; human metapneumovirus; human rhinovirus; influenza A and B; parainfluenza virus types 1–4; respiratory syncytial virus types A and B	5180	Intervention: targeted fit-tested N95 respirator	Laboratory-confirmed infection, laboratory-confirmed influenza, laboratory-detected respiratory illness, influenza-like illness, acute respiratory illness; 12wk follow-up
							Control: targeted medical mask	
Seto et al. /2003	China	Case-control study	5 hospitals: ED and medicine units	Healthcare workers	SARS	258	Intervention: N95 respirator	Laboratory-confirmed respiratory infection
							Control: surgical mask	
Zhang et al. 2013	China	Case-control study	25 hospitals: ED, respiratory wards, ICUs, outpatient departments, technical clinic departments and management	Healthcare workers	H1N1	255	Intervention: N95 respirator	Laboratory-confirmed respiratory infection
							Control: surgical mask	

A total of 8406 participants were included in the final analysis: 4355 in the N95 respirator group and 4051 in the MSM group.

### Laboratory-confirmed influenza

**Fig 2:**
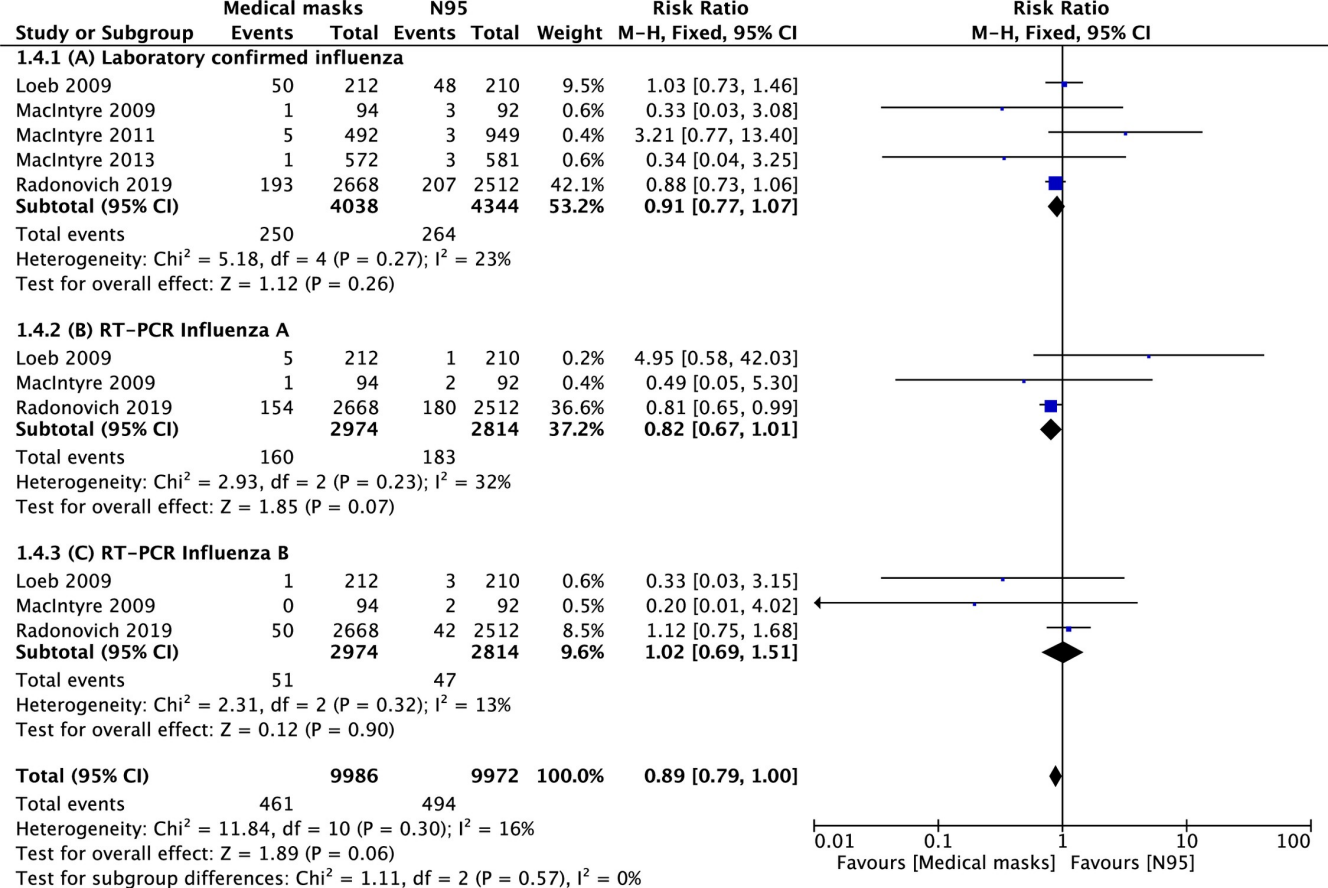
**Forest plot of:** (2A) laboratory confirmed influenza in medical masks vs. N95 groups; (2B) laboratory confirmed influenza A; (2C) laboratory confirmed influenza B. The center of each square represents the weighted mean difference for individual trials, and the corresponding horizontal line stands for 95% confidence interval. The diamonds represent pooled results.

Five RCTs reported laboratory-confirmed influenza [4–6, 8]. The pooled analysis showed that there was no statistically significant difference in laboratory-confirmed influenza between N95 and MSM (6.1 vs. 6.2%; RR = 0.91; 95% CI: 0.77–1.07; p = 0.26) (Fig 2A).

Additional analysis revealed that there were also no statistically significant differences between N95 and MSM in laboratory-confirmed influenza A risk (6.5 vs. 5.4%; RR = 0.82; 95% CI: 0.67–1.01; p = 0.07; Fig 2B) or in that of laboratory-confirmed influenza B (1.7 vs. 1.7%; RR = 1.02; 95% CI: 0.69–1.51; p = 0.90; Fig 2C).

### Laboratory-confirmed other respiratory viruses

Two RCTs presented data regarding laboratory-confirmed virus infection other than influenza [3, 4]. The prevalence of laboratory-confirmed other respiratory viruses in the N95 and MSM groups was varied and amounted to 1.9 and 1.6%, respectively (RR = 0.85; 95% CI: 0.53–1.37; p = 0.49). The sub-analyses did not show statistically significant differences in laboratory-confirmed respiratory viruses other than influenza between N95 and MSM (Table 2).

**Table 2:** Data of laboratory-confirmed other respiratory viruses in medical masks vs. N95 groups.

Parameter	No. of studies	Number of cases		RR (95%CI)	P-value	I^2^ statistic
		*Medical masks*	*N95*			
Respiratory syncytial virus	2	2/306 (0.7%)	1/302 (0.3%)	1.98 (0.18, 21.68)	0.58	NA
Metapneumovirus	2	4/306 (1.3%)	3/302 (1.0%)	1.32 (0.30, 5.83)	0.71	NA
Parainfluenza virus	2	2/306 (0.7%)	2/302 (0.7%)	0.99 (0.17, 5.67)	0.99	0%
Rhinovirus-enterovirus	2	11/306 (3.6%)	12/302 (4.0%)	0.91 (0.41, 2.02)	0.81	0%
Coronavirus	2	9/306 (2.9%)	12/302 (4.0%)	0.74 (0.32, 1.73)	0.49	NA
Adenoviruses	1	0/94 (0.0%)	2/92 (2.2%)	0.20 (0.01, 4.02)	0.29	NA
Picornoviruses	1	0/94 (0.0%)	1/92 (1.1%)	0.33 (0.01, 7.91)	0.49	NA

RR = Risk Ratio; CI = Confidence Interval; NA = Not applicable.

### Laboratory-confirmed infection with any respiratory virus

**Fig 3:**
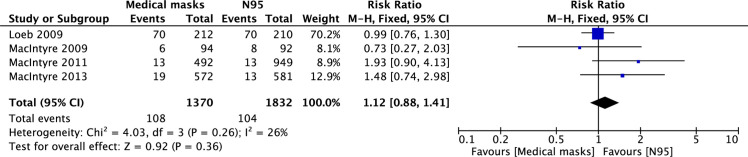
Forest plot of laboratory-confirmed infection with any respiratory viruses in medical masks vs. N95 groups. The center of each square represents the relative risk for individual trials, and the corresponding horizontal line stands for 95% confidence interval. The diamonds represent pooled results.

Laboratory-confirmed infection with any virus was reported in four RCTs [4–6]. The pooled analysis showed that N95 respirators did not reduce the risk of infection with respiratory viruses compared with MSM (5.7% vs. 7.9%; RR = 1.12; 95% CI: 0.88–1.41; p = 0.36), despite non-significant heterogeneity among the studies (I^2^ = 26%) (Fig 3).

### Laboratory-confirmed bacterial colonization

Two trials reported laboratory-confirmed bacterial colonization [6, 7], with lower risk of laboratory-confirmed bacterial colonization while using N95 compared with MSM (5.6% vs. 13.7%; RR = 2.04; 95% CI: 1.58–2.64; p < 0.001) (S3 Fig).

### Secondary outcomes

**Fig 4:**
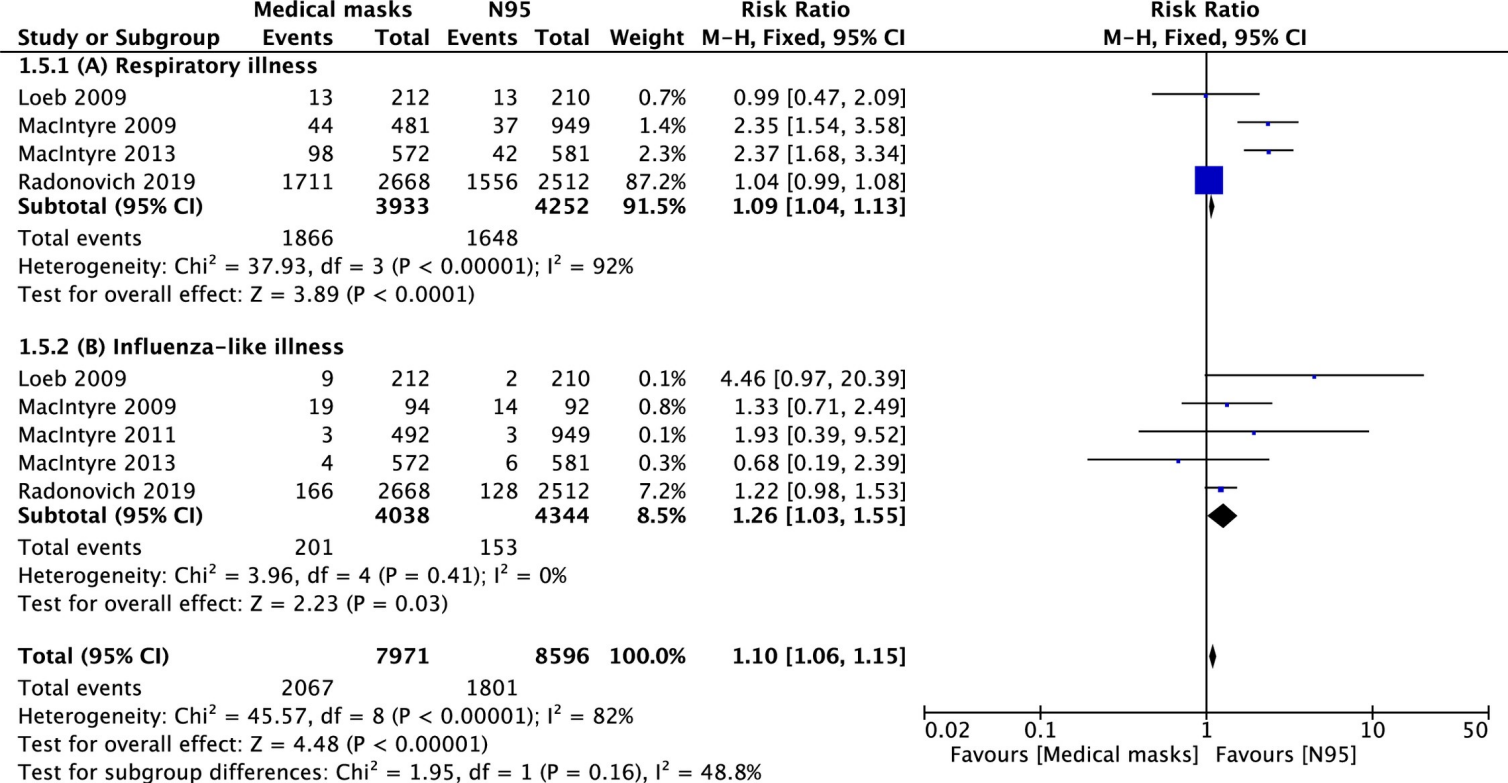
**Forest plot of secondary outcomes in medical masks vs. N95 groups:** (4A) respiratory-like illness; (4B) influenza-like illness. The center of each square represents the weighted mean difference for individual trials, and the corresponding horizontal line stands for 95% confidence interval. The diamonds represent pooled results.

Four RCTs reported respiratory illness as an outcome [4, 5, 8], with lower risk of respiratory illness in the N95 group compared with the MSM group (38.6 vs. 47.4%; RR = 1.04; 95% CI: 1.04–1.13; p < 0.001) (Fig 4A).

Influenza-like illness was observed in five RCTs [3–6, 8]. The pooled analysis showed a statistically significant difference in the risk of influenza-like illness occurrence between the N95 and MSM groups (3.5 vs. 5.0%; RR = 1.06; 95% CI: 1.03–1.55; p = 0.03; Fig 4B).

## Discussion

In this meta-analysis, we compared the use of N95 respirators with MSMs to protect against acute respiratory infections.

The rapid emergence of severe acute respiratory syndrome (SARS) due to the novel coronavirus SARS-CoV-2 (COVID-19) in December 2019 took the world by surprise. Mechanisms of transmission are believed to include contact, droplet, and possibly airborne based off of historical experiences related to SARS-CoV outbreaks [12–15]. Globally, until November 7, 2020, there have been 35,858,601 confirmed cases and 1,050,771 deaths reported in the COVID-19 pandemic [15]. As indicated by Wang et al. [16], infection prevention and control are of great importance in healthcare settings, especially with regard to personal protection of healthcare workers. Super-spreading events of SARS-CoV-2 have occurred in healthcare settings around the world. Transmission to healthcare workers within healthcare facilities has been documented with first deaths reported of physicians who acquired the disease while caring for infected patients [17]. Personal protective equipment necessary for contact with patients that are confirmed or suspected to have COVID-19 include a fluid-resistant gown, gloves, eye protection, a full-face shield, and a fit-tested N95 respirator [1, 18]. Vertical tape strips can be used to help keep gloves secured to the gown. These precautions are particularly important to emergency medicine personnel as they do not know the patients’ medical history at first contact and have no way of knowing about COVID-19 status especially with the possibility of asymptomatic transmission. Therefore, full personal protective equipment must be used when in contact with any patient.

Currently, the recommendations of the usage of masks can be contradictory as for example, the Centers for Disease Control and Prevention (CDC) recommend mask usage in both high and low risk patients; the World Health Organization recommends the usage of masks in low risk cases and respirators in high risk ones. In the current situation of a pandemic, it can be necessary for medical personal to wear N95 respirators or other masks for extended periods of time while caring for infected patients [19].

One important concern for healthcare workers is that N95 respirators need proper fit-testing to assure good seal as this is the only way to secure ambient air flow only through the filter [20]. It can occur also that multiple donning and doffing of the N95 respirators can result in test failure due to the stresses placed on the seal as the device is being removed and replaced on the face [21]. In addition, there are other concerns to the appropriate fit of the N95 respirators such as male facial hair, for example only 32% of male healthcare workers achieved adequate fit of the filtering facepiece respirator [22]. Any leakage due to an improperly installed N95 respirator potentially leads to partial breathing of ambient air without any filtration. All the analyzed studies indicated the discomfort associated with the use of N95 respirators. Many of the studies raised the issue of compliance with the recommendation to use N95 respirators or MSMs. The latter cause less discomfort, which may increase the exposure of medical personnel who put on N95 respirators without providing adequate sealing or do not use them at all recommended times [23, 24]. This factor may affect the steady effectiveness of N95 respirators in preventing acute respiratory infections. In fact, healthcare workers were able to get infected during resuscitation of patients with SARS despite wearing N95 masks [23].

## Limitations

This study has a few potential limitations. First, all RCTs included in this meta-analysis involved at least a moderate risk of bias; specifically in that the type of facemask could not be blinded to the participants in the studies. Secondly, the number of RCTs fulfilling the inclusion criteria was small. Another limitation is the fact that the results are not generalizable to infections transmitted primarily through airborne routes.

## Conclusions

Our meta-analysis suggests that there is insufficient data to definitively determine whether N95 respirators are superior to MSMs in protection against transmissible acute respiratory infections. However, we suggest N95 respirators as a more appropriate respiratory protection method than medical masks for medical personnel. Further randomized trials are necessary to compare the above methods of respiratory protection in the context of COVID-19 incidence.

## Supporting information

S1 Checklist PRISMA 2009 checklist. (DOC)

S1 Fig Evaluation of bias in all included studies across the various domains. Green, red, and yellow circles indicate low, high, and unclear risk of bias, respectively.
(TIF)

S2 Fig Summary of the risk of bias among the included studies. (TIF)

S3 Fig Forest plot of laboratory-confirmed bacterial colonization in medical masks vs. N95 groups. The center of each square represents the relative risk for individual trials, and the corresponding horizontal line stands for 95% confidence interval. The diamonds represent pooled results.
(TIF)

S4 Fig Forest plot of work absence in medical masks vs. N95 groups. The center of each square represents the relative risk for individual trials, and the corresponding horizontal line stands for 95% confidence interval. The diamonds represent pooled results.
(TIF)
