# Supplementary figures and images for: Comparative effectiveness of N95 respirators and surgical/face masks in preventing airborne infections in the era of SARS-CoV2 pandemic: A meta-analysis of randomized trials
Source: PLoS One. 2020 Dec 15;15(12):e0242901. doi: 10.1371/journal.pone.0242901 (PMC7737973; doi:10.1371/journal.pone.0242901)

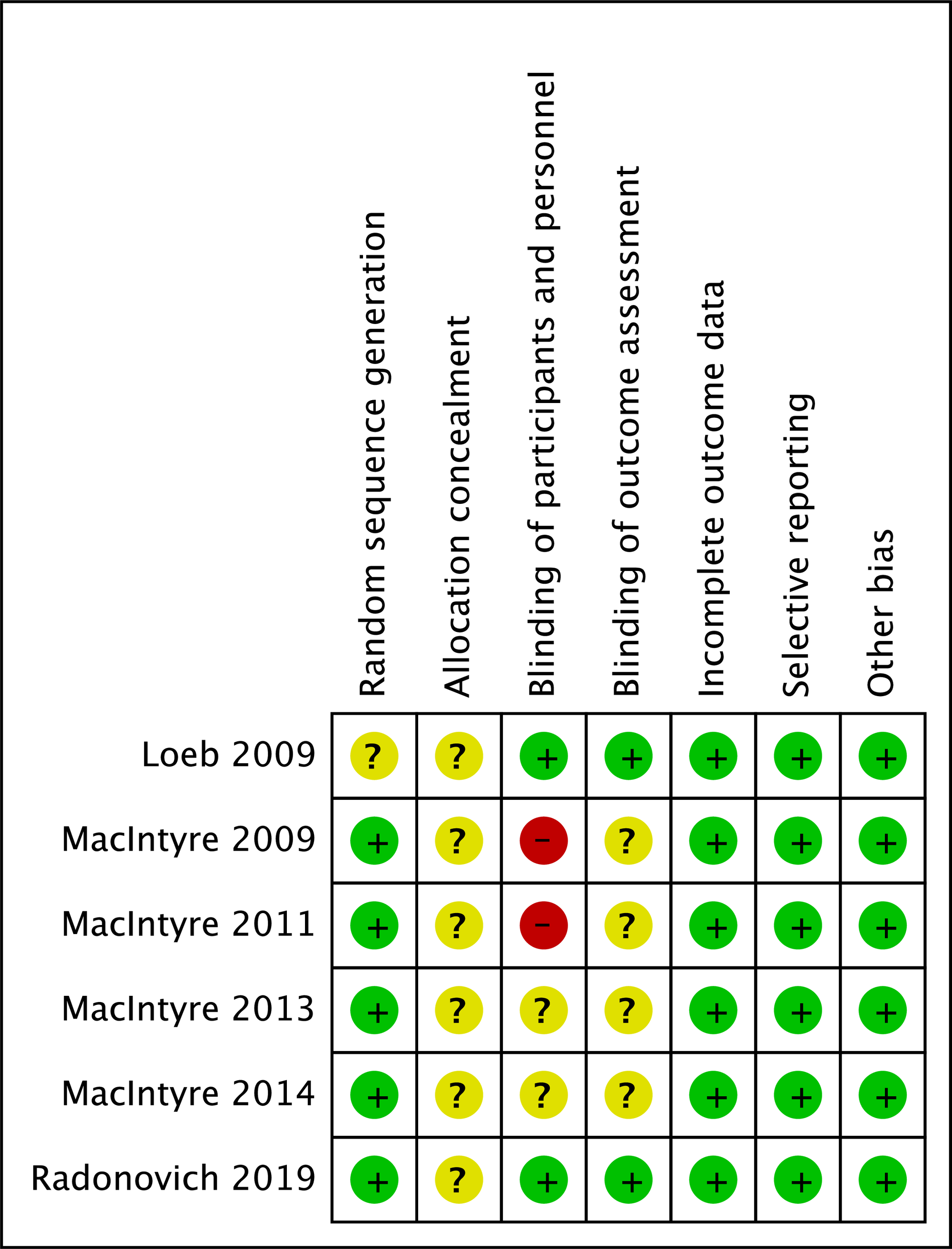

Supplement: S1 Fig — Green, red, and yellow circles indicate low, high, and unclear risk of bias, respectively. (TIF) [file pone.0242901.s002.tif]

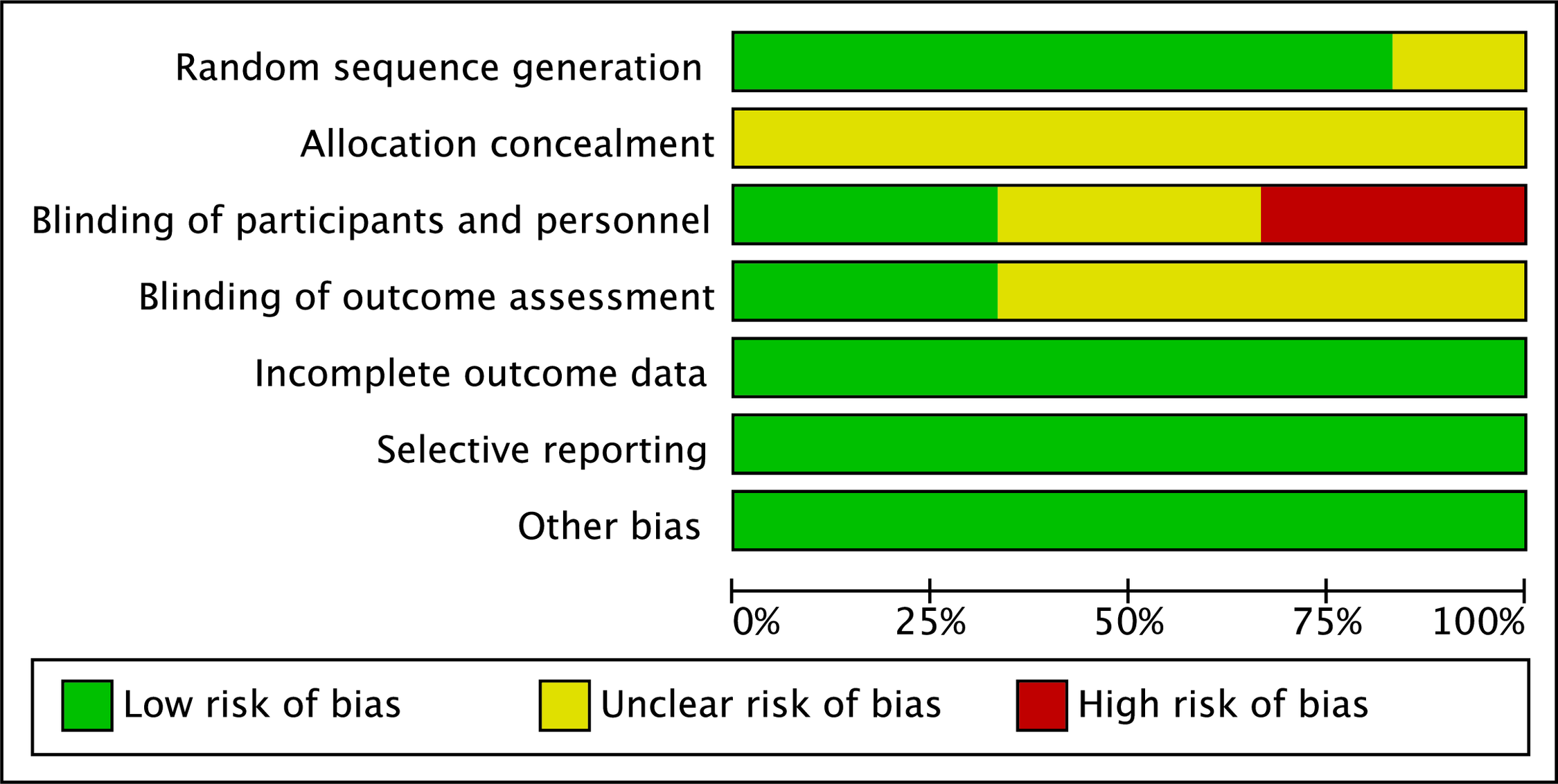

Supplement: S2 Fig — (TIF) [file pone.0242901.s003.tif]

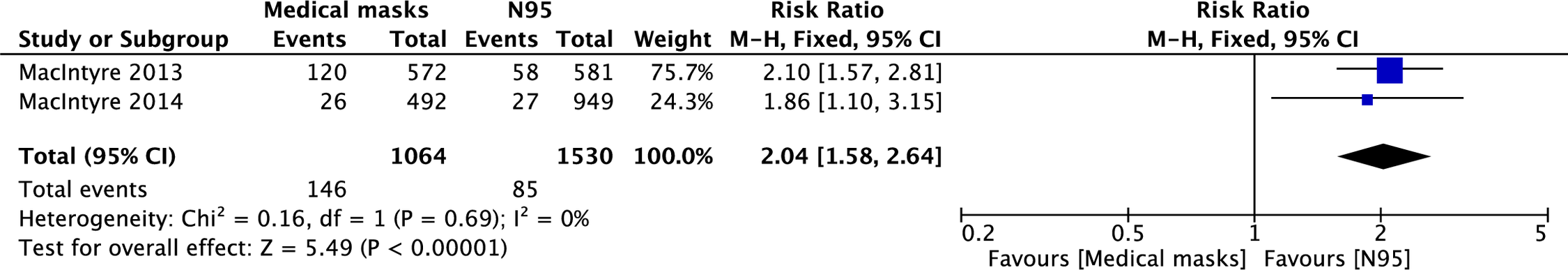

Supplement: S3 Fig — The center of each square represents the relative risk for individual trials, and the corresponding horizontal line stands for 95% confidence interval. The diamonds represent pooled results. (TIF) [file pone.0242901.s004.tif]

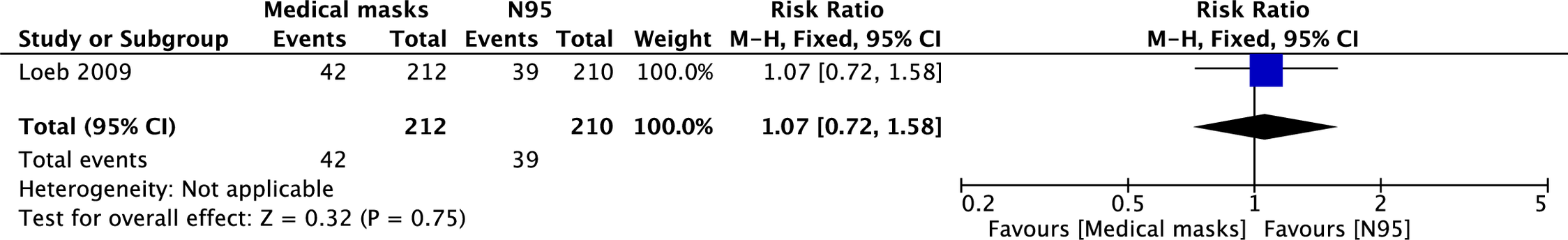

Supplement: S4 Fig — The center of each square represents the relative risk for individual trials, and the corresponding horizontal line stands for 95% confidence interval. The diamonds represent pooled results. (TIF) [file pone.0242901.s005.tif]
